# CD3^+^ T cells are critical for the resolution of comorbid inflammatory pain and depression-like behavior

**DOI:** 10.1016/j.ynpai.2020.100043

**Published:** 2020-01-21

**Authors:** Geoffroy Laumet, Jules D. Edralin, Robert Dantzer, Cobi J. Heijnen, Annemieke Kavelaars

**Affiliations:** Laboratories of Neuroimmunology, Department of Symptom Research, The University of Texas M.D. Anderson Cancer Center, Houston, TX 77030, USA

**Keywords:** T cells, CFA, Inflammatory pain, Depression-like behavior, Comorbidity, Recovery

## Abstract

**Background:**

Chronic pain and depression often co-occur. The mechanisms underlying this comorbidity are incompletely understood. Here, we investigated the role of CD3^+^ T cells in an inflammatory model of comorbid persistent mechanical allodynia, spontaneous pain, and depression-like behavior in mice.

**Methods:**

C57Bl/6 wt and *Rag2*^−/−^ mice were compared in their response to intraplantar administration of complete Freund’s adjuvant (CFA). Mechanical allodynia, spontaneous pain and depression-like behavior were assessed by von Frey, conditioned place preference and forced swim test respectively.

**Results:**

Resolution of mechanical allodynia, spontaneous pain, and depression-like behavior was markedly delayed in *Rag2*^−/−^ mice that are devoid of adaptive immune cells. Reconstitution of *Rag2*^−/−^ mice with CD3^+^ T cells from WT mice before CFA injection normalized the resolution of indicators of pain and depression-like behavior. T cells did not contribute to onset or severity of indicators of pain and depression-like behavior. The lack of T cells did not affect cytokine expression in the paw, spinal cord and brain, indicating that the delayed resolution was not resulting from prolonged (neuro)inflammation.

**Conclusions:**

Our findings show that T cells are critical for the natural resolution of mechanical allodynia, spontaneous pain, and depression-like behavior after an inflammatory challenge. Dysregulation of this T cell-mediated resolution pathway could contribute to the comorbidity of chronic pain and depression.

**Significance:**

Chronic pain and depression are frequently associated with signs of inflammation. However, general immunosuppression is not sufficient to resolve comorbid pain and depression. Here we demonstrate that T cells are required for resolution of comorbid persistent mechanical allodynia, spontaneous pain, and depression in a model of peripheral inflammation, indicating the immune system can contribute to both onset and resolution of these comorbidities. Enhancing pro-resolution effects of T cells may have a major impact to treat patients with comorbid persistent pain and depression.

## Highlights

• T cells are necessary for resolution of CFA-induced mechanical allodynia and spontaneous pain.
• T cells are required for the resolution of inflammation-induced depression-like behavior.
• T cells did not contribute to onset or severity of indicators of pain and depression-like behavior.
• T cells did not affect cytokine expression in the paw, spinal cord and brain.

## Graphical abstract

**Figure:**
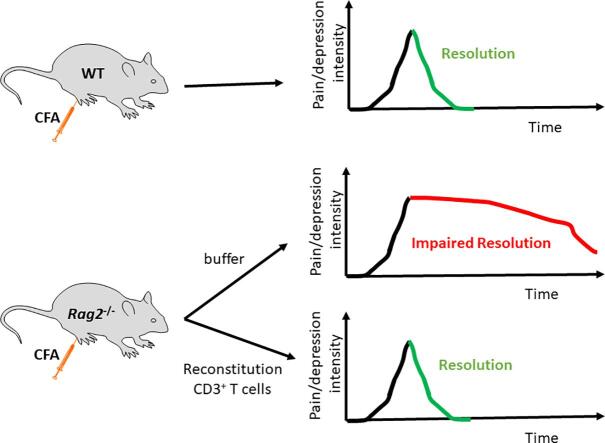

## 1 Introduction

Chronic pain is a leading health problem in North America with a lifetime prevalence of up to 20% of the U.S. and 30% of the Canadian population (Johannes et al., 2010, Schopflocher et al., 2011). Chronic pain is frequently associated with symptoms of depression (Bair et al, 2003). The relationship between chronic pain and depression is bidirectional. Depressed subjects are more likely to develop chronic pain than non-depressed individuals (Larson et al., 2004, Trivedi, 2004). Conversely, subjects suffering from chronic pain are at a higher risk for developing depression (Gustorff et al., 2008, Leo, 2005). Comorbid depression in chronic pain patients is associated with poor physical and psychosocial functioning (Holzberg et al, 1996). Both pain and depression can develop in response to inflammation (Laumet et al., 2017, Walker et al., 2014). While pain and depression are normally reversible once the initiating inflammation has resolved, pain and depression persist in some individuals. We propose that common endogenous regulatory pathways promote resolution of both pain and depression and dysregulation of these resolution pathways leads to comorbid persistent pain and depression.

Comorbidity of chronic pain and depression-like behavior develops in animal models of peripheral inflammation (Kim et al., 2012, Maciel et al., 2013). Production of proinflammatory cytokines such as tumor necrosis factor (TNF)-α and interleukin (IL)-1β by peripheral immune cells and central nervous system (CNS)-resident microglia has been proposed as a biochemical link between pain and depression (Fasick et al., 2015, Fiore and Austin, 2016, Laumet et al., 2017, Walker et al., 2014, Zhou et al., 2015). Notably, TNF-α is elevated in patients with comorbid chronic pain and depression (Bai et al., 2014, Euteneuer et al., 2011, Uceyler et al., 2007). Preclinical studies have confirmed a causal role of TNF-α and IL-1β in the onset of comorbid persistent pain hypersensitivity and depression-like behavior (Dellarole et al, 2014; Fiore and austin, 2016; Laumet et al., 2017, Maciel et al., 2013, Norman et al., 2010, Zhou et al., 2015).

An emerging body of literature indicates a critical contribution of peripheral immune cells to not only the onset, but also the resolution of pain and depression-like behavior (Baddack-Werncke et al., 2017, Bang et al., 2018, Brachman et al., 2015, Cohen et al., 2006, Ji et al., 2011, Labuz et al., 2010, Willemen et al., 2014). In particular, we recently showed that chemotherapy-induced mechanical neuropathic pain (Krukowski et al., 2016, Laumet et al., 2019a) and lipopolysaccharide (LPS)-induced depression-like behavior (Laumet et al, 2018) were markedly prolonged in mice devoid of adaptive immune cells. Reconstitution of immunodeficient mice with T cells was sufficient to normalize the resolution indicating, that T cells are necessary not only for resolution of chemotherapy-induced mechanical neuropathic pain (Krukowski et al., 2016, Laumet et al., 2019a) but also for resolution of LPS-induced depression-like behavior (Laumet et al, 2018).

To further study the role of T cells in the resolution of both comorbid pain and depression-like behavior, we now used the model of intraplantar injection of complete Freund’s adjuvant (Kim et al., 2012, Maciel et al., 2013) in WT and *Rag2*^−/−^ mice that are devoid of adaptive immunity. The role of T cells was studied by reconstituting *Rag2*^−/−^ mice with T cells from WT mice before injection of CFA. In addition, we assessed the mRNA expression of *Tnf* and *Il1β* in tissue relevant for mechanical allodynia, spontaneous pain, and depression-like behavior.

## 2 Methods

### 2.1 Animals

Male WT and *Rag2*^−/−^ mice (9–12 weeks old) in a C57Bl/6 background (Jackson laboratory, Bar Harbor, ME) were maintained in the animal facility of The University of Texas MD Anderson Cancer Center. Mice were housed in a reverse light cycle (light off 8:00 am and light on 8:00 pm) and were randomly assigned to group. Peripheral inflammation was induced by administration of 5 µg CFA (1 mg/ml, each ml of CFA contains 1 mg of heat-killed and dried Mycobacterium tuberculosis, 0.85 ml paraffin oil and 0.15 ml of mannide monooleate. Sigma) injected into the plantar surface of the left hind paw. Control mice received an equi-volume injection of saline. All procedures were approved by the Institutional Animal Care and Use Committee (IACUC) and in accordance with NIH guidelines for the care and use of animals. All analyses were performed by investigators blinded to treatment and genotype.

### 2.2 CD3^+^ T cells isolation and adoptive transfer

Adoptive transfer of CD3 + T cells to *Rag2*^−/−^ mice was performed 10 days before CFA or saline injection as previously described (Krukowski et al., 2016, Laumet et al., 2018, Laumet et al., 2019a). Spleens were collected from WT mice and single cells suspensions were obtained by passing spleens though a 70 µm mesh. CD3^+^ T cells were isolated using a negative selection kit II (#130–095-130, Miltenyi Biotec Inc, San Diego, CA). Eight million CD3^+^ T cells were intravenously (i.v.) injected into the tail in a 100 µl volume. Control mice received an i.v. injection of PBS-BSA. Homing and survival of the adoptively transferred cells were confirmed by flow cytometry as previously described (Krukowski et al., 2016, Laumet et al., 2018, Laumet et al., 2019a). Briefly, blood cells were stained with anti-CD45-APC, anti-CD3-PE, anti-CD4-Cy5.5 and anti-CD8-FITC (BD bioscience #561018, #561799, #550954 and #553031, San Jose, CA) antibodies. Lysing buffer was used to remove red blood cells. Samples were analyzed with the C6 Accuri (BD Biosciences). We gated leukocytes based on CD45 expression followed by gating on CD3 + and then CD4 + and CD8 + cells to identify subsets of T cells.

### 2.3 Behavior

Mechanical allodynia was quantified using the von Frey calibrated filaments. The mechanical stimulus producing a 50% likelihood of withdrawal was determined using the “up-down” calculating method as previously described (Chaplan et al., 1994, Garriga et al., 2018, Laumet et al., 2015).

Spontaneous pain was measured using the conditioned place preference (CPP) paradigm as previously described (King et al., 2009, Krukowski et al., 2017, Laumet et al., 2019a, Vichaya et al., 2018, Yang et al., 2014). The CPP apparatus consisted of 2 chambers (18 × 20 cm, one dark, one bright) connected by a 15 cm hallway (Stoelting, Wood Dale, IL). On the first day each mouse freely explored the apparatus for 15 min. Conditioning took place over 4 days. In the morning, mice were injected i.p. with phosphate buffered saline (PBS) and were individually placed 10 min later in the dark chamber for 15 min. Four hours later, mice were injected i.p. with 10 mg/kg of the analgesic drug retigabine i.p. (#R-100, Alomone laboratory, Jerusalem, Israel) and placed in the bright chamber. On the sixth day drug free mice explored the apparatus for 15 min and the change in time spent in the bright (previously analgesic-paired) chamber was quantified. The pre- and post-conditioning tests were recorded and analyzed using video tracking software (Noldus Ethovision XT).

Depression-like behavior was quantified as increased immobility in the forced swim test (FST). Mice were placed in a bucket (19 cm in diameter, 29 cm high) filled with water (25 ± 1C) and immobility time was recorded for the last 5 min of a 6 min trial as previously described (Laumet et al., 2018, Laumet et al., 2017).

Because alterations in locomotor activity can bias the tests used for measuring depression-like behavior and conditioned place preference, spontaneous locomotor activity was recorded. Individual mice were placed in a 50 × 30 × 30 cm new cage. The distance traveled was recorded for 5 min and quantified by video tracking software (Noldus Ethovision XT).

Behavioral testing was performed and scored by experimenters blinded to experimental group treatment.

### 2.4 Gene expression

Mice were terminated with C02 procedure. The ipsilateral paw, ipsilateral lumbar spinal cord (SC), and contralateral prefrontal cortex (PFC) were rapidly removed and snap frozen in liquid nitrogen. RNA were isolated and gene expression analyzed as previously described (Laumet et al., 2015, Laumet et al., 2017). Briefly, total RNA was extracted from the tissues using the Trizol/chloroform method. cDNA was prepared by using the high capacity cDNA reverse transcription kit (#4368813, Applied Biosystems, Foster City, CA). The quantitative PCR was performed using the CFX-384 real-time system (Biorad, Hercules, CA) and gene were amplified with the PrimeTime standard qPCR assay (IDT DNA technologies, Coralville, Iowa). The relative amount of *Il1b* and *Tnf* genes in each sample was first normalized to the level of a housekeeping gene, *Gapdh*, and then normalized to its expression level in saline-treated mice.

### 2.5 Statistical analysis

Data are presented as mean ± SEM. Statistical difference between 2 or multiple groups was determined by *t*-test, one-way, or two-way ANOVA depending on the experimental design followed by Bonferonni’s multiple comparison tests when needed. Statistical analysis was performed using GraphPad Prism 6.0 (La Jolla, CA).

## 3 Results

### 3.1 T Lymphocytes promote resolution of inflammatory pain

**Fig. 1:**
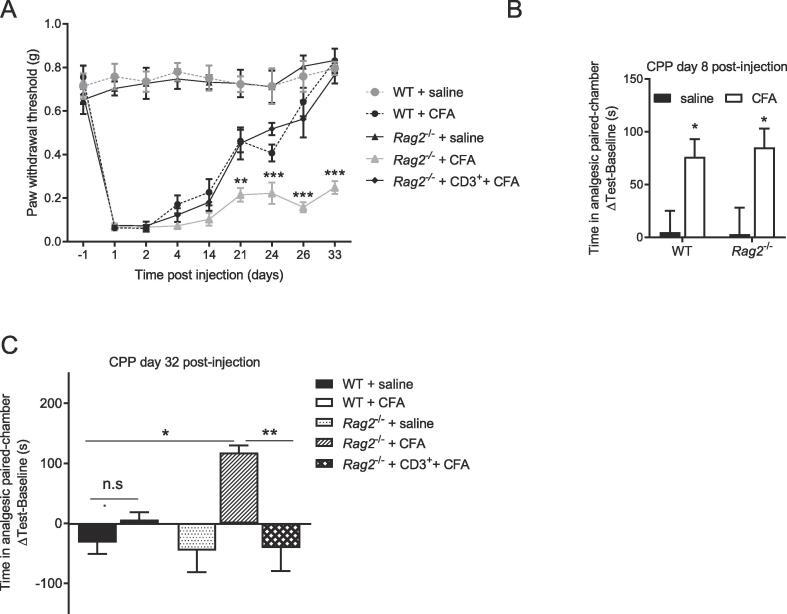
Effects of T cells on the duration of pain in response to Complete Freund’s Adjuvant (CFA). A) Mechanical pain sensitivity was monitored in WT, *Rag2*^−/−^ and reconstituted *Rag2*^−/−^ mice treated with saline or CFA (n = 8 mice/group). Repeated measures two-ways ANOVA followed by Bonferonni’s correction (time × genotype interaction, F (32, 264) = 11.4, p < 0.0001. ** = p < 0.01 and *** = p < 0.001 comparing *Rag2*^−/−^ + CFA vs. *Rag2*^−/−^ + CD3^+^ + CFA. B) Difference in time spent in the analgesic-paired chamber before (baseline) and after (test) conditioning in WT and *Rag2*^−/−^ mice 8 days after intraplantar injection of saline or CFA (n = 8/group). Two-way ANOVA followed by Bonferonni’s correction (CFA × genotype interaction, F(1, 28) = 0.07, p = 0.79; main factor CFA, F(1,28) = 14.3, p = 0.0008). C) Difference in time spent in the analgesic-paired chamber before (baseline) and after (test) conditioning in WT, *Rag2*^−/−^ and reconstituted Rag2−/− mice 32 days after intraplantar injection of saline or CFA (n = 8/group). One-way ANOVA followed by Bonferonni’s’s correction (F=(4,35) = 6.75, p = 0.0004). Significant statistical difference was indicated by * = p < 0.05, ** = p < 0.01 and *** = p < 0.001. Data are presented as mean ± standard error of the mean.

In WT mice, injection of CFA induced mechanical allodynia that lasted 21 days (Fig. 1A, n = 8/group). In *Rag2*^−/−^ mice, CFA-induced allodynia persisted for at least for 33 days (Fig. 1A). Reconstitution of *Rag2*^−/−^ mice with CD3^+^ T cells before CFA administration normalized the resolution of allodynia (Fig. 1A). The initial severity of mechanical allodynia in response to CFA was similar in all groups. All groups had similar paw withdrawal thresholds at baseline (Fig. 1A).

**Fig. 2:**
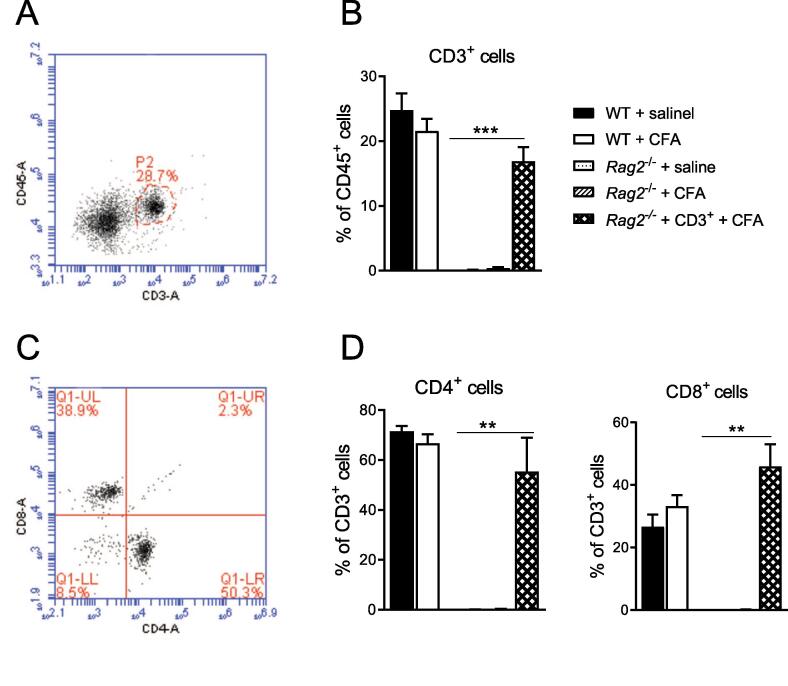
Representative plots illustrating the gating strategy. A) Representative flow cytometry plots of CD3 expression on CD45 + cells. B) Percentage of circulating CD45^+^ CD3^+^ cells measured by flow cytometry 38 days after intraplantar saline or CFA injection (n = 5 mice/group). C) Representative flow cytometry plots of CD4 and CD8 expression on CD3 + cells isolated from WT 38 days after CFA injection. D) Percentage of circulating CD3^+^CD4^+^ and CD3^+^CD8^+^ cells measured by flow cytometry 38 days after intraplantar saline or CFA injection (n = 5 mice/group). Significant statistical difference was indicated by * = p < 0.05, ** = p < 0.01 and *** = p < 0.001. Data are presented as mean ± standard error of the mean.

Clinically, spontaneous pain is a more important problem than mechanical allodynia (Vierck et al, 2008). To measure spontaneous pain, we used the conditioned place preference (CPP) paradigm. The development of a marked preference for the analgesic-paired chamber indicates that mice experience spontaneous pain in response to CFA. Starting the conditioning on day 3 post-injection and testing the mice on day 8, we observed that WT and *Rag2*^−/−^ mice had developed similar preference for the analgesic-paired chamber in response to CFA (Fig. 1B, n = 8/group). To determine whether spontaneous pain is prolonged in the absence of T cells, we performed an independent experiment in which we started the conditioning on day 28 (test on day 32). At 33 days after CFA, WT mice no longer displayed a preference for the analgesic-paired chamber, indicating that the spontaneous pain had resolved at this time point (Fig. 1C, n = 8/group). In contrast, CFA-treated *Rag2*^−/−^ mice still developed a preference for the analgesic-paired chamber (Fig. 1C). *Rag2*^−/−^ mice reconstituted with CD3 + T cells behaved like WT mice (Fig. 1C). These findings indicate that CD3 + T cells are required for the resolution of spontaneous pain in response to CFA. T cell reconstitution of the *Rag2*^−/−^ mice was confirmed by flow cytometry of peripheral blood (Fig. 2, n = 5/group).

### 3.2 T Cells promote recovery from CFA-induced depression-like behavior

**Fig. 3:**
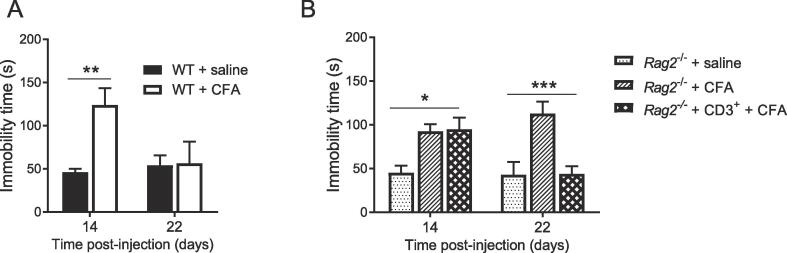
Effects of T cells on the duration of depression-like behavior in response to CFA. A) The forced-swim test was performed 14 d and 22 d after intraplantar injection of CFA or saline in WT mice (n = 6 mice/group). Two-way ANOVA (time × treatment interaction, F(1,20) = 4.85, P = 0.04). (B) The forced-swim test was performed 14 d and 22 d post-injection in *Rag2*^−/−^ and reconstituted *Rag2*^−/−^ mice (n = 7 mice/group). Two-way ANOVA followed by Bonferroni’s correction (T cells × treatment interaction, F(2,18) = 7.11, P = 0.005). The FST was performed in the same mice on day 14 and 22. Significant statistical difference were indicated by * = p < 0.05, ** = p < 0.01 and *** = p < 0.001. Data are presented as mean ± standard error of the mean.

Next, we investigated depression-like behavior as measured in the FST. In WT mice, CFA administration increased immobility time in the FST as assessed at 14 days post-CFA. The increased immobility time was no longer apparent 22 days post-CFA, indicating resolution of depression-like behavior in WT mice (Fig. 3A, n = 6/group). In contrast, *Rag2*^−/−^ mice still displayed increased immobility at 22 days post-CFA (Fig. 3B, n = 7/group). However, when *Rag2*^−/−^ mice had been reconstituted with CD3^+^ T cells, depression-like behavior had resolved at 22 days after CFA. The CFA-induced increase in duration of immobility in the FST 14 days post-CFA was similar in WT and *Rag2*^−/−^ mice.

**Fig. 4:**
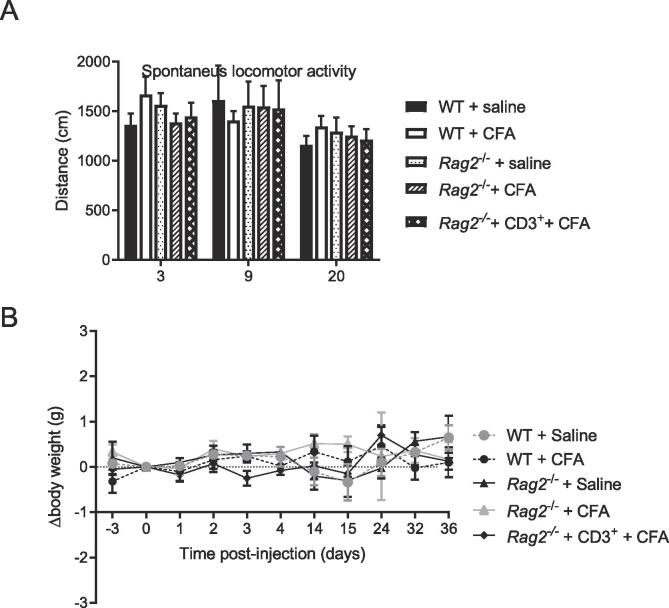
Effects of T cells and CFA on measures of sickness behavior. A) Spontaneous locomotor activity was assessed 3, 9 and 20 days after intraplantar CFA or saline -injection in WT, *Rag2*^−/−^, and reconstituted *Rag2*^−/−^ mice (n = 4–5 mice/group). B) Body weight was monitored over time in WT, *Rag2*^−/−^, and reconstituted *Rag2*^−/−^ mice after CFA or saline injection (n = 4–5 mice/group).

Peripheral inflammation may induce sickness behavior which may overlap with depression-like behavior. To assess whether prolonged depression-like behavior in *Rag2*^−/−^ mice was associated with prolonged or more intense sickness we monitored body weight and spontaneous locomotor activity (Laumet et al, 2018). CFA injection had no effect on body weight (<1 g change) and spontaneous locomotor activity in WT, *Rag2*^−/−^ mice, and in *Rag2*^−/−^ mice reconstituted with T cells (Fig. 4).

### 3.3 T Cells do not affect *Tnf* and *Il1*b expression after CFA

**Fig. 5:**
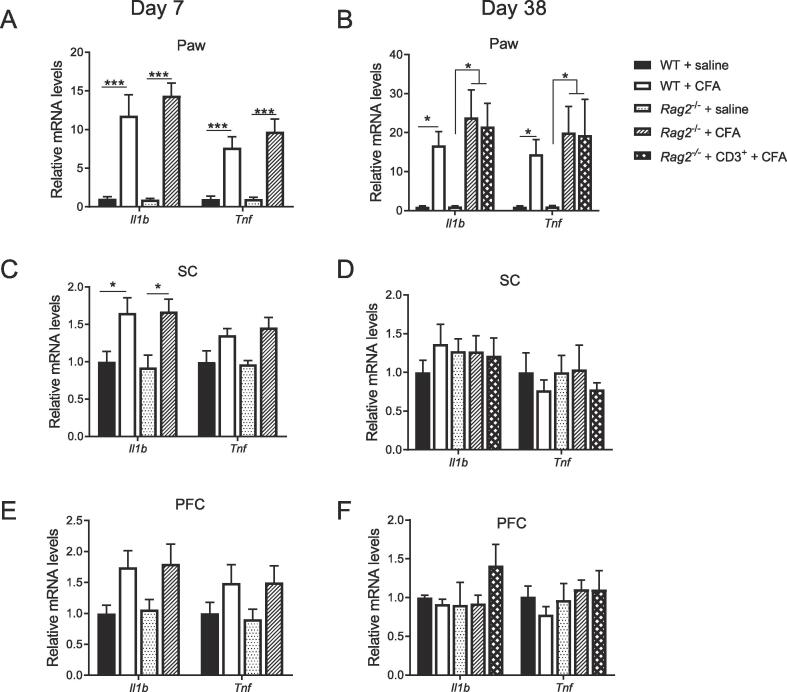
Effects of T cells on peripheral and neuroinflammation in response to CFA. *Tnf* and *Il1b* mRNA expression in A,B) ipsilateral paw, C,D) ipsilateral lumbar spinal cord (SC), and E,F) contralateral prefrontal cortex (PFC) at 7 and 38 days after intraplantar injection of CFA or saline (n = 5–8 mice/group). Two-way ANOVA followed by Bonferroni’s correction with CFA as a main factor, A) F(3, 36) = 37.6, P < 0.0001; B) F(4,40) = 8.8.4, P < 0.0001; C) F(3, 46) = 10.5, P < 0.0001; E) F(3, 46) = 5.16, P < 0.0037. Genotype × CFA interactions were not statistically significant for any groups. Significant statistical difference was indicated by * = p < 0.05 and ** = p < 0.01. Data are presented as mean ± standard error of the mean.

To determine whether the prolonged allodynia, spontaneous pain, and depression-like behavior observed in T cell-deficient mice result from more pronounced or prolonged (neuro)inflammation, we measured mRNA expression of the prototypical pro-inflammatory cytokines *Il1b* and *Tnf* at the site of CFA injection (paw), and in the CNS (ipsilateral lumbar spinal cord (SC) and contralateral prefrontal cortex (PFC)). At 7 days after CFA, *Il1b* and *Tnf* were robustly upregulated in the paw but cytokine expression did not differ between WT and *Rag2*^−/−^ mice (Fig. 5A). At 38 day post-CFA, *Il1b* and *Tnf* mRNA levels were still upregulated in the paw in WT, *Rag2*^−/−^ and reconstituted *Rag2*^−/−^ mice, but there were no group differences (Fig. 5B). SC levels of *Il1b* were increased in both genotypes at 7 days post-CFA (Fig. 5C, *Il1b*: WT + saline (n = 7) vs, WT + CFA (n = 6) p = 0.016; *Rag2*^−/−^ + saline (n = 7) vs. *Rag2*^−/−^ + CFA (n = 6) p = 0.04; *Tnf*: WT + saline (n = 7) vs, WT + CFA (n = 7) p = 0045; *Rag2*^−/−^ + saline (n = 7) vs. *Rag2*^−/−^ + CFA (n = 7) p = 0.10) and back to baseline at 38 days (Fig. 5D). *Tnf* was not significantly upregulated in the SC (Fig. 5D). In the PFC, CFA increased *Il1b* and *Tnf* expression in WT and *Rag2*^−/−^ mice at 7 days and cytokine levels were no longer elevated at 38 days (Fig. 5E, *Il1b*: WT + saline (n = 8) vs, WT + CFA (n = 6) p = 0.16; *Rag2*^−/−^ + saline (n = 6) vs. *Rag2*^−/−^ + CFA (n = 7) p = 0.19; *Tnf*: WT + saline (n = 8) vs, WT + CFA (n = 6) p = 0.81 ; *Rag2*^−/−^ + saline (n = 6) vs. *Rag2*^−/−^ + CFA (n = 7) p = 0.49). An overall CFA effect was apparent (p = 0.004) at 7 days post-CFA (Fig. 5E, F). There were no group differences in PFC cytokine mRNA levels (Fig. 5E,F). These findings indicate that the prolonged allodynia, spontaneous pain, and depression-like behavior in T cell-deficient mice did not result from differences in acute or persistent upregulation of *Tnf* or *Il1b* expression in paw, spinal cord and PFC.

## 4 Discussion

The present findings demonstrate for the first time that CD3^+^ T cells are required for the resolution of comorbid persistent mechanical allodynia, spontaneous pain, and depression-like behavior in response to peripheral inflammation. Mechanical allodynia, spontaneous pain and increased immobility time in the FST were significantly prolonged in *Rag2*^−/−^ mice which do not have T and B cells, compared to WT mice. Reconstitution of *Rag2*^−/−^ mice with CD3 + T cells before CFA administration was sufficient to normalize the resolution of both pain and depression-like behavior in Rag2−/− mice.

Accumulating preclinical evidence indicates a critical role of T cells in promoting the resolution from neurological disorders. The absence of T cells delays the resolution of pain (Baddack-Werncke et al., 2017, Boue et al., 2011, Krukowski et al., 2016, Laumet et al., 2019a) For example, chemotherapy-induced peripheral neuropathy is strikingly prolonged in the absence of T cells and reconstitution with T cells prevents the development of chronic pain. Likewise T cells are critical to promote resolution of inflammation- or stress-induced depression-like behavior (Brachman et al., 2015, Cohen et al., 2006, Laumet et al., 2018). The neuroprotective role of T cells is not limited to nociception and depression, it has been shown in models of nerve injury as well (Jones et al, 2015). One limitation is that most of these studies involved only male mice. Given the sex difference observed in neuro-immune interactions, further studies using female mice are necessary to address potential sex difference in the pro-resolution effect of T cells.

Our findings indicate that a T cell dysfunction may contribute to the comorbidity of pain and depression. This notion is supported by clinical evidence. For example, patients with irritable bowel syndrome (IBS) who present with depressed symptoms and persistent pain, have a lower number of circulating T cells than healthy controls (Swiatkowski and Rybakowski, 1993). Reductions in circulating T cells and in mitogen-induced T cell proliferation have been reported in patients with symptoms of depression (pain was not assessed in these studies) (Grosse et al., 2016, Leday et al., 2017, Miller, 2010, Snijders et al., 2016, Toben and Baune, 2015).

*Rag2*^−/−^ mice do not have T and B cells and we show it is sufficient to reconstitute these mice with T cells to normalize resolution of inflammatory pain and depression-like behavior. Thus, our findings indicate B cells are not required for onset, severity, or resolution of inflammatory pain and depression-like behavior following CFA injection. Nevertheless, it is possible that B cells participate in behavioral alteration and neuroinflammation induced by CFA. Additionally, it is likely that T cells interact with other immune cells such as macrophages to promote resolution (Laumet et al., 2019b). Indeed, we showed earlier that macrophages are required for the resolution of pain (Bang et al., 2018, Krukowski et al., 2016, Willemen et al., 2014).

In our CFA model, the prolonged mechanical allodynia, spontaneous pain, and depression-like behavior observed in *Rag2*^−/−^ mice is not mediated by prolonged or exaggerated inflammation at the site of CFA injection and in the CNS. At 7 days after CFA, *Tnf* and *Il1b* expression in the paw, lumbar spinal cord and brain were similar in WT and *Rag2*^−/−^ mice. These observations are in line with previous studies from us and others where similar expression of proinflammatory cytokines were observed in the brain in WT and *Rag2*^−/−^ mice in response to LPS (Clark et al., 2015, Laumet et al., 2018). These data indicate that resolution of inflammation is unlikely to be sufficient to resolve pain and depression-like behavior. Likewise, resolution of pain and depression-like behavior in the WT is not associated with full resolution of paw inflammation. This dissociation between the resolution of inflammation on the one hand and pain and depression on the other hand is supported by clinical data. For example, effective treatment of inflammation in patients with rheumatoid arthritis or inflammatory bowel syndrome is not always associated with the resolution of pain (Bielefeldt et al., 2009, Lee et al., 2011, Lomholt et al., 2013). Our data suggest that pharmacological treatment of comorbid pain and depression with anti-inflammatory drugs like NSAIDs will not be sufficient to resolve the pain and depression. Our data indicate that functional T cells (and/or their products) are necessary to treat the symptoms of pain and depression. Our current findings might also explain that inhibition of proinflammatory cytokine signaling (e.g., anti-TNF-α) has a limited effect on major depressive disorders (Kappelmann et al., 2018, Raison et al., 2013).

A potential mechanism for the T cells to promote resolution of mechanical allodynia, spontaneous pain and depression-like behavior is the release of endogenous opioids. T cells produce endogenous opioids in response to peripheral inflammation and this reduces allodynia (Baddack-Werncke et al., 2017, Basso et al., 2016, Basso et al., 2018, Boue et al., 2011; (Labuz et al., 2009); Labuz et al., 2010, Lutz and Kieffer, 2013, Maestroni and Conti, 1991, Pecina et al., 2019). It has been proposed that this endogenous opioid production suppresses neuronal activity to counterbalance the pro-nociceptive effects of cytokines, we predict that the resolution mechanisms in reconstituted *Rag2*^−/−^ mice are similar to those in WT mice. Therefore, reconstituted *Rag2*^−/−^ mice should be in a state of latent sensitization after resolution of allodynia, as has been described for WT mice (Corder et al, 2013). Whether T cells indeed release endogenous opioids under the conditions described here to induce resolution of comorbid pain and depression would require further investigations.

## 5 Conclusion

Chronic pain and depression often occur together. Both can result from inflammation and activation of the innate immune system and the release of cytokines. However, the mechanisms that underlie the resolution of comorbid pain and depression are unknown. Our present findings add to a growing body of literature (Baddack-Werncke et al., 2017, Brachman et al., 2015, Duffy et al., 2019, Filiano et al., 2017, Krukowski et al., 2016, Laumet et al., 2018, Laumet et al., 2019a) demonstrating that CD3^+^ T cells are necessary for the resolution of comorbid pain and depression-like behavior after peripheral inflammatory. Interestingly, the presence of T cells did not affect the expression of proinflammatory cytokines in the brain, the spinal cord, and at the site of injection. These data indicate that T cells promote resolution of comorbid mechanical allodynia, spontaneous pain, and depression-like behavior independently of the resolution of neuroinflammation and peripheral inflammation even when inflammation is the original trigger. The exact mechanism by which T cells promote the resolution of comorbid mechanical allodynia, spontaneous pain, and depression-like behavior in response to peripheral inflammation still needs to be elucidated. From a clinical perspective, our findings are important because they indicate that T cell dysfunction may contribute to the persistence of comorbid pain and depression.

Our data contribute to understanding why anti-inflammatory therapies will not be sufficient to effectively treat pain and depression because T cell dependent active resolution pathways have to be engaged for full recovery.

## CRediT authorship contribution statement

**Geoffroy Laumet:** Investigation, Formal analysis, Writing - original draft. **Jules D. Edralin:** Investigation. **Robert Dantzer:** Conceptualization, Writing - review & editing. **Cobi J. Heijnen:** Conceptualization, Writing - review & editing. **Annemieke Kavelaars:** Conceptualization, Writing - review & editing, Supervision.
